# Metastatic adenocarcinoma within a functioning adrenal adenoma: a case report

**DOI:** 10.4076/1757-1626-2-7965

**Published:** 2009-07-02

**Authors:** Jeremiah T Martin, Fuad Alkhoury, Scott Helton, Paul Fiedler, Olga Sakharova, Steven Yood

**Affiliations:** Department of Surgery, Hospital of St. Raphael1450 Chapel Street, New Haven, CT 06511USA

## Abstract

We present the case of a 54-year-old woman who underwent right adrenalectomy for palliation of Cushing’s symptoms. She had recently been diagnosed with lung adenocarcinoma. Pathologic findings revealed a 5 cm adrenal adenoma with a metastatic adenocarcinoma deposit. The occurrence of tumor-to-tumor metastasis is rare, and the finding of a metastasis within a functional adrenal adenoma exceptionally so. Previously reported incidences of this finding in patients with lung cancer range from 0.14% to 0.63%. We review the literature regarding this unusual finding.

## Introduction

Tumor-to-tumor metastasis is an infrequent occurrence. Here we report on the finding of metastatic lung adenocarcinoma within a functioning adrenal adenoma which was excised for palliation of Cushing’s symptoms in a 54-year-old female.

## Case presentation

**Figure 1.**
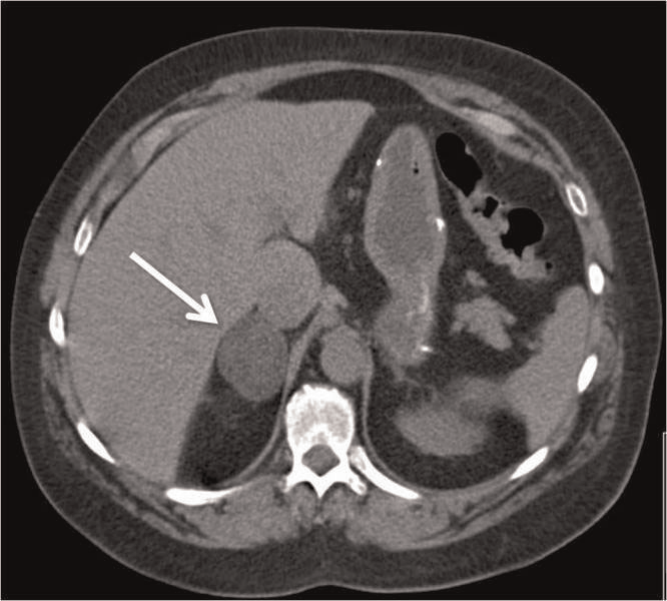
Abdominal CT scan, arrow depicts right adrenal mass.

A 54-year-old Caucasian American female who previously had a hiatal hernia repair was referred for abdominal CT scan after operative findings of hepatomegaly were noted. On CT (Figure 1) and subsequent MRI a 4 cm right adrenal adenoma with benign radiologic characteristics was noted. Over the preceeding two years the patient had symptoms consistent with Cushing’s syndrome with complaints of weight gain, lower extremity swelling and muscle weakness. Workup revealed an elevated free urinary cortisol (195 µg/day), suppressed ACTH (<5 pg/mL) consistent with adrenal Cushing’s syndrome. She was scheduled for laparoscopic adrenalectomy, however a liver lesion was found at laparoscopy which, on biopsy, was positive for metastatic adenocarcinoma of pulmonary origin. The procedure was terminated at this point for further patient counseling. Upon the patient’s insistence, she was again scheduled for adrenalectomy to control her Cushing’s symptoms prior to undergoing therapy for her lung cancer.

**Figure 2.**
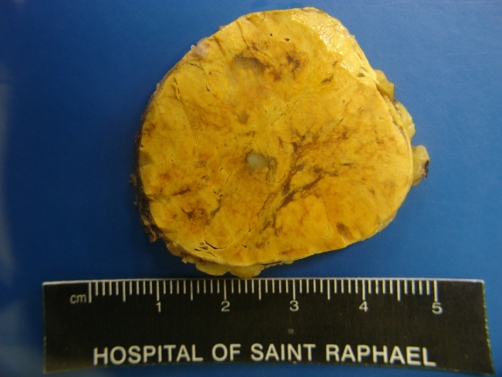
Gross Photo shows yellow adrenal cortical adenoma with small, gray focus slightly left of center representing metastatic adenocarcinoma.

**Figure 3.**
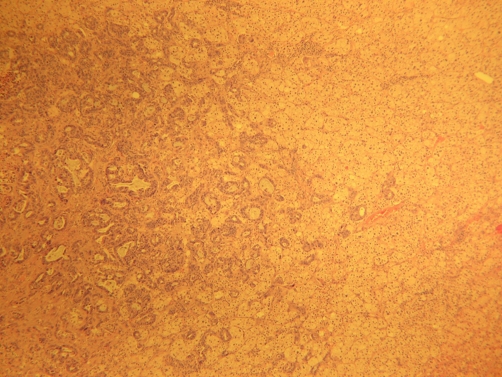
Photomicrograph shows metastatic adenocarcinoma on the left and adrenal cortical adenoma on the right (H&E stain - Low Power).

**Figure 4.**
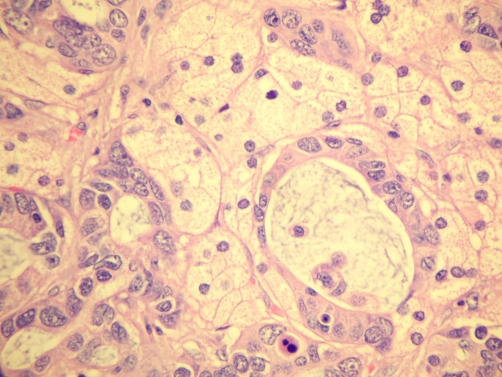
Photomicrograph shows malignant glands (adenocarcinoma) intermixed with foamy adrenal cortical adenoma cells (H&E stain - High Power).

**Figure 5.**
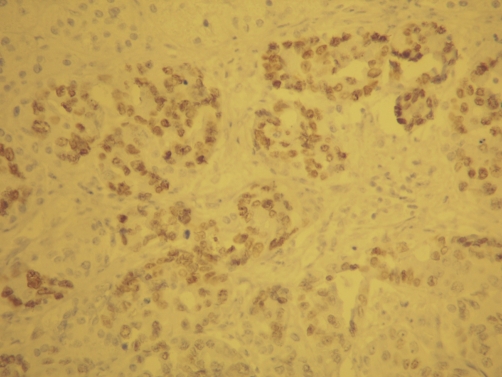
TTF-1 immunostain demonstrates nuclear positivity of adenocarcinoma, consitent with lung origin.

**Figure 6.**
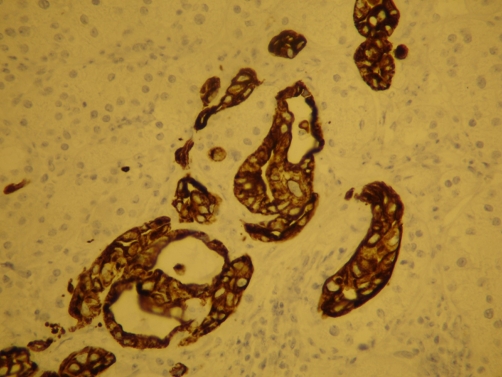
Cytokeratin 7 immunostain demonstrates cytoplasmic positivity of adenocarcinoma, consistent with lung origin.

At laparoscopy, a 5 × 4 × 4 cm, 45 g right adrenal mass was identified and removed without complication (Figure 1). The patient recovered well and was commenced on supplementary corticosteroids. Final pathology revealed an adrenal cortical adenoma with a focus of adenocarcinoma, positive for cytokeratin 7 and TTF-1, an immunophenotype consistent with lung primary (Figures 2-6).

## Discussion

The occurrence of adrenal metastasis in the setting of non-small cell lung cancer (NSCLC) is not uncommon. In patients with NSCLC, the incidence has been reported from 25-40% during the course of the disease [1,2]. Adrenal metastases likely develop via lymphatic spread in early disease and via hematogenous spread in more advanced disease. This is evidenced by a greater propensity towards ipsilateral metastasis early in the disease course, with contralateral or bilateral metastases more likely to occur with advanced disease [2].

Despite the relative frequency of adrenal metastasis, this finding in the setting of an adrenal adenoma is rare. This phenomenon has been studied by Moriya et. al who noted a 0.63% incidence of metastasis to an adrenal adenoma in a review of lung cancer autopsy cases [3]. This compares with an incidence of 0.14% found by Onuigbo et al. in a review of 7232 lung cancers [4]. The statistical analysis of Moriya’s group indicated that there was a propensity for lung cancer to metastasize to an adrenal adenoma if present.

Tumor-to-tumor metastasis is an uncommon occurrence, and has only been documented in sporadic case reports and series since 1902 [5]. Fewer than 100 cases have been reported in the literature. Our institution previously reported a case of a colonic adenocarcinoma metastasizing to a thyroid adenoma [6]. The phenomenon generally involves metastasis from a donor (malignant) tumor to either a benign or malignant recipient tumor. Lung cancer is the most common primary tumor involved in up to 50% of the reported cases [7]. Other donor tumors which have been reported include breast, gastrointestinal, prostate and thyroid malignancies. The most frequent benign recipient is meningioma [8] with renal cell carcinoma being the most common malignant recipient [9].

Generally, the presence of an adrenal metastasis in the setting of lung cancer classifies the patient as having Stage IV disease and therefore benefiting most from chemotherapy. There is some evidence that patients with otherwise surgically resectable lung cancer and documented isolated adrenal metastasis may benefit from simultaneous resection [10]. This diagnosis can be difficult to determine preoperatively as it is far more likely for a screening CT scan to reveal an incidental adenoma. That said, thorough inspection of any adenoma with chemical shift imaging on MRI can with some confidence determine whether or not a mass is completely benign [11]. Given the high incidence of adrenal metastasis over the course of NSCLC, regular screening should include careful follow up of any adrenal masses. Metachronous adrenal masses should be resected when there is suspicion of adrenal metastasis in the setting of adrenal enlargement or cytologic confirmation [12].

The authors are not aware of other cases of NSCLC metastasis to a functioning adrenal adenoma. Tumor-to-tumor metastasis has been reported in the setting of a non-functioning adenoma [11]. The primary indication for adrenalectomy in our patient was to palliate her from Cushing’s symptoms. Following resection of her tumor she underwent adjuvant therapy for her lung cancer.

Early identification of adrenal metastases is essential as adrenalectomy may improve survival in selected patients. To this end, physicians caring for patients with lung cancer must maintain a high index of suspicion to detect these lesions.

## Abbreviations

ACTH: Adrenocorticotrophic hormone
NSCLC: Non small-cell lung cancer
TTF-1: Transcription termination factor 1
